# Hardware assisted hypervisor introspection

**DOI:** 10.1186/s40064-016-2257-7

**Published:** 2016-05-17

**Authors:** Jiangyong Shi, Yuexiang Yang, Chuan Tang

**Affiliations:** College of Computing, NUDT, Deya Road, Changsha, 410072 China; Information Center, NUDT, Deya Road, Changsha, 410072 China

**Keywords:** VMI, Hypervisor introspection, Hypercall interception, Nested virtualization, Security

## Abstract

In this paper, we introduce hypervisor introspection, an out-of-box way to monitor the execution of hypervisors. Similar to virtual machine introspection which has been proposed to protect virtual machines in an out-of-box way over the past decade, hypervisor introspection can be used to protect hypervisors which are the basis of cloud security. Virtual machine introspection tools are usually deployed either in hypervisor or in privileged virtual machines, which might also be compromised. By utilizing hardware support including nested virtualization, EPT protection and #BP, we are able to monitor all hypercalls belongs to the virtual machines of one hypervisor, include that of privileged virtual machine and even when the hypervisor is compromised. What’s more, hypercall injection method is used to simulate hypercall-based attacks and evaluate the performance of our method. Experiment results show that our method can effectively detect hypercall-based attacks with some performance cost. Lastly, we discuss our furture approaches of reducing the performance cost and preventing the compromised hypervisor from detecting the existence of our introspector, in addition with some new scenarios to apply our hypervisor introspection system.

## Introduction

Virtual machine introspection (VMI) is an out-of-box way to monitor the virtual machine (VM) execution. It is firstly proposed by Garfinkel and Rosenblum (2003). As no agents are needed to be installed in VMs, VMI are more stealthy than traditional host security tools such as Firewall and Anti-virus software. A lot of security applications based on VMI have been proposed over the past decade (Jiang et al. 2007; Srivastava and Giffn 2008; Nance et al. 2009). However, the precondition of VMI’s functioning is the security of hypervisor, where VMI tools are deployed.

Just like system calls (syscall, for short) may be used by attackers to escalate privileges, hypercalls can be used by attackers to break virtualization’s isolation and escape virtual machines’ constraints and finally attack the hypervisors. For example, CVE-2015-2045 allows local guest users to obtain sensitive information via unspecified vectors. As HInjector (Milenkoski et al. 2013) summaries, hypercall injection attacks can be classified into three types, namely invoking hypercalls from irregular call sites, invoking hypercalls with anomalous parameter values and invoking a series of hypercalls in irregular order. Besides these misuse cases, there are also vulnerabilities in hypercall handlers, which are studied in detail based on practical CVE vulnerabilities (Milenkoski et al. 2014a, b), some of which are also repeated by reverse engineering the patches and creating POC codes. These vulnerabilities are divided into two types: implementation errors which include value validation errors and incorrect implementation of inverse procedures, and non-implementation errors. Besides, for open source hypervisors such as Xen and KVM, it is possible that the source code is being tampered and stealthy malicious hypercalls are being added as backdoors to attackers. Even though there is no real such hypercall-based attack being reported, that doesn’t mean it is not important or it doesn’t exist. Maybe it is because the current security tools are unable to detect this advanced type of attack.

**Fig. 1:**
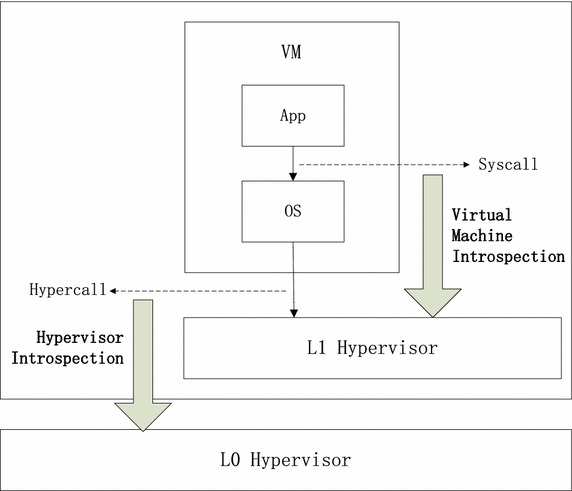
Comparison of HVI and VMI

To detect hypercall-based attacks, we extended the concept of VMI and proposed a new concept similar to VMI, namely hypervisor introspection (HVI). The main goal of HVI is to extract hypercalls from out of hypervisor so as to monitoring the execution of guest VMs. This is crucial in detecting hypercall-based attacks. Because hypercalls are initialized by guest kernels, it is hard to detect it in guests. Although we can deploy the detector in dom0 to detect hypercalls of domU, dom0 could also be compromised and utilized to issue the malicious crafted hypercalls. That’s why we use nested hypervisors to deploy our detectors as shown in Fig. 1. By implementing hypercall detectors in the lowest hypervisor (L0), we are able to detect all hypercalls initialized from the guests in the top hypervisor (L1), despite of this from dom0 or domU.

There are a lot of advantages using nested hypervisor to study the behavior of hypervisor. Firstly, it requires no additional hardware support except for the nested virtualization support, which is fully supported by almost all the recent CPUs from Intel and AMD. Secondly, it can be easily adjusted to monitor different versions of L1 hypervisors. As the monitoring tool is under the lowest layer (L0), we can easily update the monitor to adapt new version of hypervisors. Thirdly, compared with other solutions, our hypervisor monitor is more stealthy with proper anti-detection methods which will be discussed in detail in “Discussion and future work”.

In particular, our work mainly makes the following contributions:

- We describe the implementation details of our hypervisor introspection system which provides two methods to intercept hypercalls and detect hypercall-based attacks.
- We improve the accuracy of hypercall intercepting by #BP and distinguishing normal #BP from injected one.
- We improve the performance of hypercall intercepting by setting intervals in EPT protection.
- We introduce a hypercall sequence analyzing method based on hypercall injection and Stide method.
- We discuss hypervisor anti-detection mechanisms so as to prevent the attackers from discovering the detector’s existence.

The remainder of this paper is organized as follows. Some backgrounds about nested virtualization and hardware features are introduced in “Background”. The framework of our hypervisor introspection system is described in “Design”. In-depth details about our implementation are presented in “Implementation”. The experiments using recent hypercall injection technology are described in “Analysis”. Our future works of performance improving, anti-detection of L0 hypervisor and possible application scenarios are discussed in “Discussion and future work”. Related works are discussed in “Related work” and a brief conclusion is made finally.

## Background

### Nested virtualization

Nested virtualization mainly includes nested virtualization of CPU and nested virtualization of memory.

Nested virtualization of CPU is firstly implemented by binary translation of privileged instructions. With the support of new CPU feature, including Intel VMX and AMD SVM, nested virtualization of CPU becomes easier and more efficient. Binary translation can reduce the time cost when there is a TLB miss, but hardware assisted virtualization can more efficiently support privileged instructions. So, hardware assisted virtualization is better in CPU intense workload while binary translation is better in memory intensive workload. As the hypercalls are more concerned with executing privileged CPU instructions, we choose hardware assisted virtualization as our solution.

Nested virtualization of memory can be divided into software-based and hardware-based. Software-based nested virtualization is by extending the shadow page table and adding another layer of memory translation, while hardware-based nested virtualization makes use of the hardware feature support, which is named as EPT by Intel and NPT by AMD (McDougall and Anderson 2010). As the nested shadow page is very inefficient, we are mainly concerned with hardware-based one.

Running PV guests as an L2 has been supported in Xen since the introduction of HVM guests in Xen 3.0 (He 2009). However, support for HVM guests as L2 guests is heavily dependent on architecture-specific support. It is not until 2012 when Intel upgrades nested virtualization support with virtual EPT and virtual VT-d that nested HVM guests are fully supported (Zhang and Dong 2012). With virtual EPT support, guest memory address can be directly translated to machine address using shadow EPT in L0 hypervisor, instead of two times of translation from virtual address to address in L1 then to address in L0. This greatly improved the performance and universality of nested virtualization.

### EPT protection and #BP

There are a lot of reasons which cause VMEXIT as listed in Intel (2015). By utilizing these features we can effectively capture VM events like hypercalls. Among these features, EPT Violation and Breakpoint Exception (#BP) are most common and easy to use.

With EPT and virtual EPT, we are able to set access permissions for memory pages. For example, we can set a page only writable and executable to hypervisor or dom0, but readable to domU. Any writes from a domU would cause an EPT violation and VMEXIT. By intercepting the VMEXIT events, we are able to find the write behavior which might be a signature of attack. However, EPT protection operates on a page granularity, which means one hypercall function may cause multiple EPT violations during its execution. This would introduce high performance cost. And that’s why we consider #BP in the same time.

#BP is widely used in debugging. Improperly use of it might cause the OS crash, so executing it on a VM would cause VMEXIT. By manually inject 0xCC into hypercall functions, it will trigger #BP whenever the hypercall functions are called, thus capturing hypercalls from outside of hypervisor. Actually, this is right exactly how we use it to intercept hypercalls. Even though it will still cause VMEXIT and increase the execution time, it is more accurate and efficient than the EPT Protection method, which will be verified in “Analysis” section.

## Design

### Assumptions and requirements

Our development of HVI conforms to the following requirements and assumptions:

- No modifications to the hypervisor and no additional agents or clients are installed in the VMs of the monitored hypervisor.
- The hypervisor might be compromised and malicious, but the attackers don’t have physical access to hardware resources.
- Advanced attacks which use higher privileges than hypervisor are not considered. Such attacks include SMM-based rootkits (Embleton et al. 2008), BIOS based rootkits or EFI based Bootkits (Kleissner 2009; Hudson and Rudolph 2015), DMA attacks through peripherals (Wojtczuk 2008) and even Chipset-based rootkits (Tereshkin and Wojtczuk 2009). The countermeasures need a combination of hardware architecture enhancement (Szefer and Lee 2012) and management education to prevent the attackers from accessing the hardware, which is out of our scope.
- A bottom hypervisor (L0) is more secure and robust than the top hypervisor (L1). As we use L0 to monitor hypercalls from L1, it is possible that the compromised top hypervisor might try to discovery and tamper with the bottom hypervisor. Although there are methods trying to prevent the L1 from discovering the L0, it still has remote possibility that the L1 might find new ways to detect the existence of L0. This assumption can be practical by using different hypervisors or newer edition of hypervisors as L0. For example, we can use a latest Xen as L0 to monitor old version of Xen, so as to discover new attacks to old products. Usually latest products have fewer bugs compared with old ones. So the L0 can be considered more secure than L1. As old version products always coexist with the latest one, this is very useful in studying the behavior of attacks to hypervisors. Moreover, we can simplify the L0 hypervisor or customize one as we only need the specific function related with hypercall intercepting, thus reducing the TCB of the L0. This is very similar to what Trustvisor does (McCune et al. 2010), only that Trustvisor is a tiny nested hypervisor used to protect the VMs of L1, while we want to protect L1.
- We assume that the L0 has enough resources to be used to monitor L1. So during the design and implementation of our system, we adhere to the principle of minimizing the impact to L1 by executing the most heavy job on L0. The smaller impact on L1, the fewer chance of our system’s being detected.
- Hardware virtualization support is needed to implement our system, specially CPUs with Intel VT-x and EPT features (or SVM and NPT for AMD CPUs). During our implementations, we use the latest nested virtualization technique of virtual EPT to improve the performance of L1 and enable EPT protection. In our experiment, the CPU is E5-2603 v3 which meets our requirements.

### Architecture

**Fig. 2:**
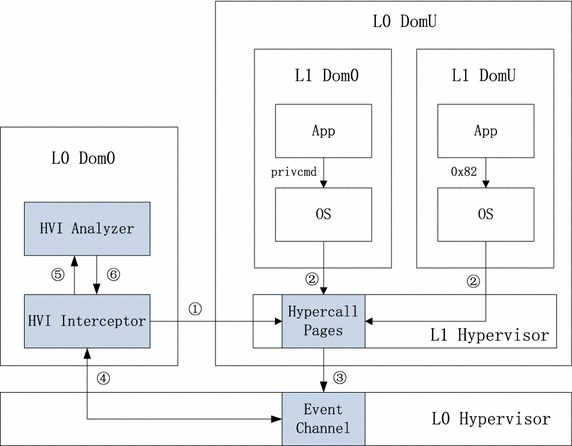
Architecture of hypervisor introspection system

The main architecture of our HVI system is shown in Fig. 2. It is based on nested virtualization and mainly consists of two parts: HVI Interceptor and HVI Analyzer.

The Interceptor takes two methods to intercept the hypercalls of L1. The first one is to inject 0xCC into hypercall functions and intercept #BP caused by these hypercalls. It communicates with the L0 hypervisor through XenControl Library (libxc) to obtain #BP caused VMEXIT events. The breakpoint injection process is completed with help of LibVMI, a C library capable of viewing the L1 hypervisor’s memory, trapping on hardware events, and accessing the vCPU registers (Payne 2012). The second method is by utilizing EPT protection feature of Intel CPU. By setting execution protection on hypercall page, any calls to hypercall will induce an EPT violation and cause the L1 enter to VMEXIT. Then we can handle the VMEXIT in L0 and obtain the information related with hypercalls.

The Analyzer receives hypercall related information from the Interceptor, such as hypercall number and hypercall arguments. The analyzing method can be various, such as comparing the k-size sequence with normal sequences and decide if it’s malicious based on the total number of mismatch (Maiero and Miculan 2011), or that based on Markov and Bayesian models (Wu et al. 2014). Here we use the method provided in Maiero and Miculan (2011) for its simplicity and effectiveness in syscall analyzing.

Nested hypervisor is used so as to do HVI. The L0 hypervisor may differ from the L1 hypervisor and is suggested to do so because if the L1 hypervisor is vulnerable, the L0 hypervisor could also be subverted if they remained the same. It will greatly improve the difficulty of subverting them simultaneously as L0 hypervisor’s security can be enhanced using physical protection and tidy design. However, it should be noticed that nested hypervisors need the support of hardware, such as Intel’s EPT or AMD’s NPT, which are supported by most recently CPUs.

The use of Xen hypercalls is different between PVM and HVM, and different between user-level applications and kernel. PVM read hypercall page through interrupt 0x82 while HVM access hypercall page by reading registers using CPUID instructions. Guest user-level application cannot access hypercall page directly. Instead, they use ioctl to manipulate a special file named privcmd under */proc/xen* directory, so as to indirectly access the hypercall page. No matter how the user-level application and the kernel use hypercalls, they would finally come to call their entries at hypercall page. So in order to keep generality, we directly monitor the execution or hypercall pages, instead of user-level hypercall interfaces.

We use a nested Xen to implement our system. The workflow of our system is shown in Fig. 2. First, the Interceptor write traps (0xCC) to the hypercall functions of L1 hypervisor through LibVMI, which has control over the L1 domU through Libxc (Step 1). Then the hypercalls from L1 hypervisor guests (Step 2), whether PVM or HVM, no matter dom0 or domU, will trigger the breakpoints and cause VMEXIT (Step 3). Then the VMEXIT events will be delivered to HVI through event channels (Step 4). After obtaining the events, the HVI Analyzer could collect the hypercall related information, such as hypercall number and hypercall arguments (Step 5). Based on the analyzing results of these hypercalls, the HVI can take response actions, such as write back the original value to get it through, or drop it if it’s malicious (Step 6).

## Implementation

### Locating of hypercalls

Similar to syscall, hypercalls deliver arguments using generic registers. Among them, %eax is used to store the hypercall number, while other registers such as %ebx, %ecx, %edx, %esi and %edi are used to store hypercall related arguments. We can monitor these registers to get hypercall related information. However, as these registers are general purposed and can be used by all system applications and other system functions, the results would be very nosiy and the performance cost would be very high. Instead, we use another method which is similar to syscall hooking. The basic idea is to set traps at hypercall page so as to cause interruption when the functions are called.

Refer to syscall hooking, we need firstly locate the hypercall page for the VM to be monitored. The initialization of hypercall page is accomplished by function *hypercall_page_initialise* during the process of domU and dom0 creating. A hypercall page is divided into 128 blocks, each of which contains the code to initialize a hypercall (entries). Currently, there are only 40 general hypercalls and 8 architecture-specific hypercalls. As every block is 32B large, we can calculate every hypercall’s virtual address by adding 32 times hypercall number to the hypercall page base address. And the hypercall page’s virtual address can be obtained from the symbol file of dom0.

### Hypercalls intercepting

Because of the existence of hypervisor, there is an additional address translation during the process of guest virtual address to physical machine address. So we need to translate the virtual address of hypercall page into machine address to set traps and monitor all the hypercalls. This can be done with LibVMI, an open source virtual machine introspection library. LibVMI translates the L1 address of hypercall entries into L0 addresses. The basic idea of the approach is utilizing the *xc_map_foreign_range* function, provided by XenControl Library (libxl).

After we get the physical address of hypercall page, we can set traps on hypercall entries deemed of interest by adding the base address of hypercall page with hypercall number times of 32B, which is the size of hypercall entry. The process of setting traps is the core part. First, we inject a breakpoints (0xCC) in the place of hypercall entries. Then we configure the CPU to issue a VMEXIT when breakpoints are hit and configure Xen to forward these events to L0 hypervisor. This technique (#BP) has been used in probing (Quynh and Suzaki 2007; Carbone et al. 2014), debugging (Deng et al. 2013) and VMI (Lengyel et al. 2014; Estrada et al. 2015). As far as we know, this is the first time of applying it to hypercall intercepting and hypervisor protecting.

One problem when implementing #BP injection is that we have to differentiate the normal 0xCC breakpoints from the injected ones. Because 0xCC is usually used to debug, improperly handler of it may cause the guest crash. Our policy is by comparing the address where #BP happens with the addresses of hypercall entries. If it is within one of the hypercall entries, we record the useful information and write back the original value so that the hypercall can execute successfully. If it is out of the range of the hypercall page, we just write the 0xCC back to let it behave normally.

Another approach we take is by setting EPT execute protection on hypercall page, which has been applied in protecting user mode processes (Lutas 2015). But to the best of our knowledge, this is the first time to use EPT protection to intercept hypercalls. EPT protection can only set on a page unit, which fits well to hypercall page. However, as every hypercall entry’s size is 32B which contains multiple instructions, one hypercall would cause multiple EPT violations, thus causing performance degradation. To solve that, we analyzed the code in hypercall page initialization function (shown below) and find that there are multiple instructions for every entry when the hypercall page is initialized. So we wait the CPU execute some steps before reseting the EPT protection bits. This would prevent the same hypercalls from repeatedly triggering the EPT violation and theoretically reduce the performance cost, which will be demonstrated in the “Analysis” section.

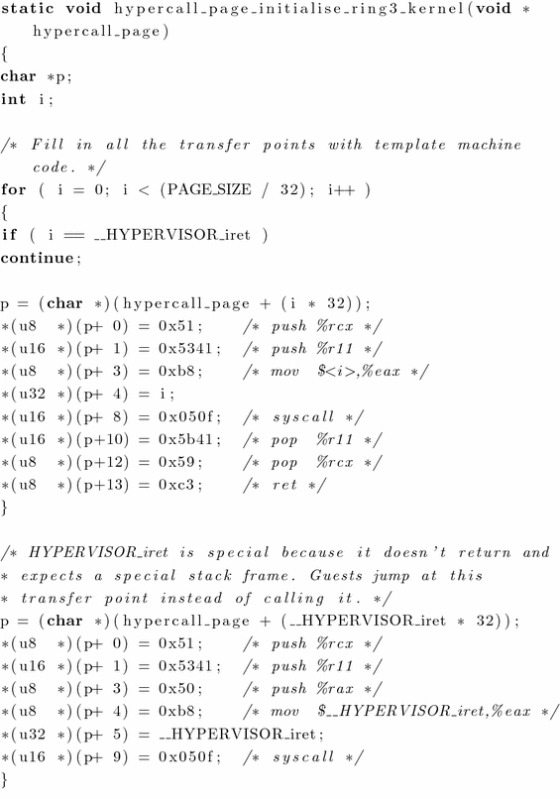



### Hypercalls analyzing

To analyze the hypercalls and detect hypercall-based attacks, we should first collect normal samples and malware samples. Normal samples can be collected by running legal applications on guests of L1 and be used to train the detector. However, malware samples which use hypercall-based attacks are rare in reality, which makes a big obstacle. Luckily, the latest work named HInjector (Milenkoski et al. 2015) solved the problem. In order to issue hypercalls as we need, HInjector inserts a module into the guest kernel and use xml format to configure the hypercalls. In the Xen hypervisor, HInjector can identify the hypercalls we craft and choose to block its execution for security reasons. This is perfect to test our system as we need the crafted malicous hypercalls to verify if our Interceptor can capture these hypercalls and if our Analyzer can identify these anomalies based on analysis.

**Fig. 3:**
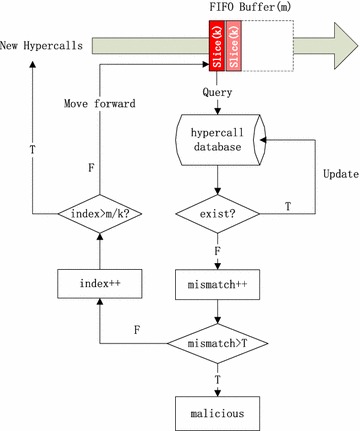
Working flow of HVI analyzer

To analyze the intercepted hypercalls, we consider using syscall analyzing method. Stide (Forrest et al. 1996) is chosen for its simplicity and efficiency in evaluating syscall sequences (Maiero and Miculan 2011). The basic structure of HVI Analyzer is shown in Fig. 3.

When a new hypercalls is intercepted by Interceptor and delivered to Analyzer, the Analyzer would combine it with the former $k-1$ hypercalls to be a *k*-size slice. Then the slice is sent into a FIFO buffer whose size is *m*. After that, the Analyzer queries the hypercall database where benign hypercall sequences are stored to see if the slice exists. If not, the mismatch number will increase, otherwise the hypercall database will be updated and the hit sequence count increases. When the mismatch in greater than the threshold (T), the hypercall sequence in the buffer will be recognized as malicious, other wise the slice index will increases and move forward to next slice in the buffer until all slices are queried. When all slices are detected and the mismatch is still below the threshold, the hypercall sequence in the buffer will be recognized as normal and new hypercalls are accepted for analysis.

This method is simple and resource saving. For a hypercall sequence of length n, there is at most 6*mn*/*k* instructions need to be executed. As 6*m*/*k* is a constant, the complexity is *O*(*n*), which is very suitable in real-time monitoring. While other advanced machine learning methods need to do complex computing whose complexity may be $O(n^{2})$ or even more. Besides, we can also store the hypercalls into disk and do the analysis offline when there is idle resource, which is very common in cloud service.

### Improving HVI-EPT

**Fig. 4:**
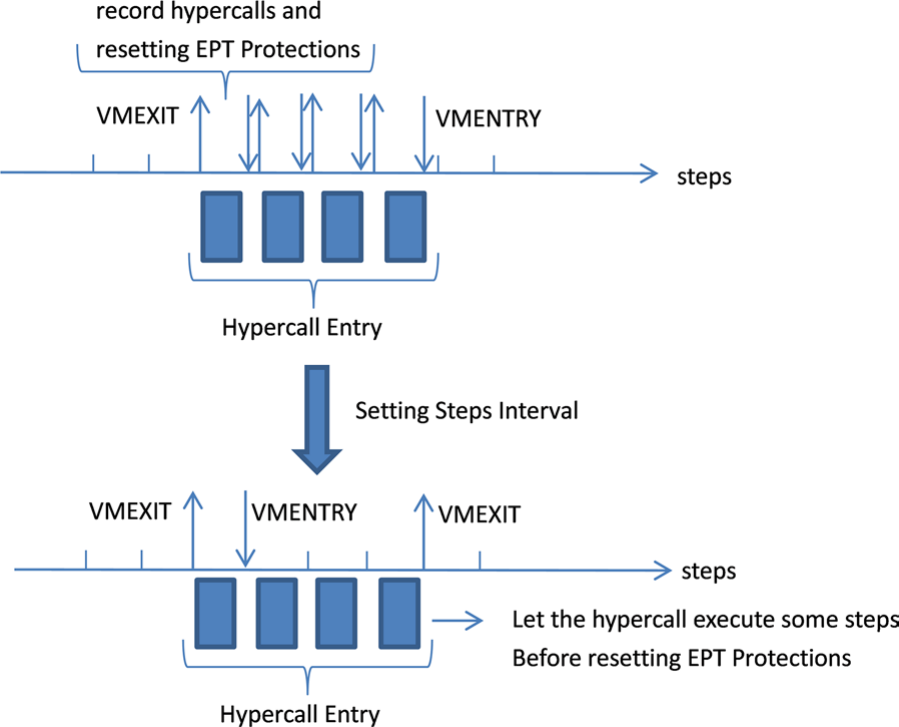
One hypercall would cause multiple VMEXITs with HVI-EPT

When using HVI-EPT, a step setting between reinjection of breakpoints is very small granularity to an assembly instruction. Shown in Fig. 4, there are multiple steps for each hypercall entry. If we just reinject 0xCC during each VMEXIT caused by HVI-EPT, one hypercall would cause four VMEXITs and the records would be duplicated. In order to reduce the performance cost of HVI-EPT, we randomize the steps to reinject traps after a EPT violation is handled, so as to reduce the VMEXIT events.

**Fig. 5:**
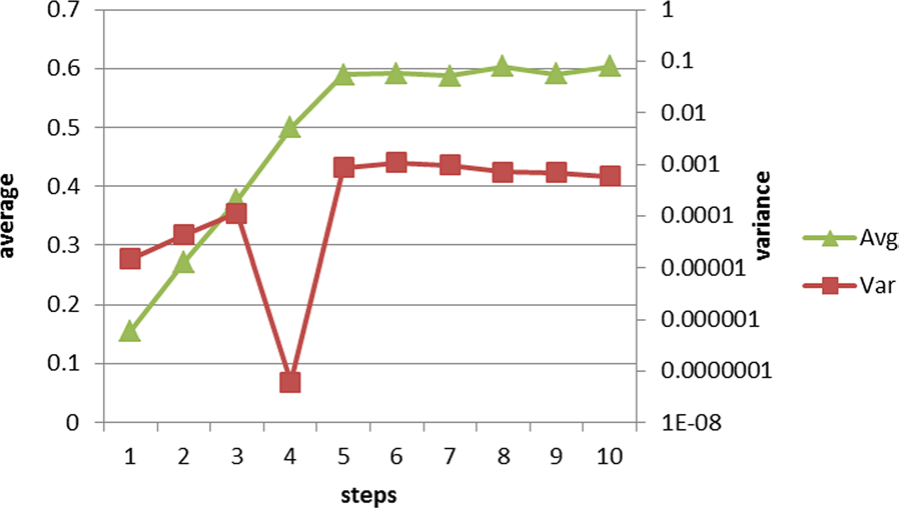
The rate of hypercalls to EPT violations when setting different steps between VMEXITs

When the steps are set smaller than 4, the L1 is still during the process of hypercall caused VMEXITs, thus cannot utilize this interval as it is paused during the VMEXIT. When the steps are set larger than 4, it might miss some hypercalls and cause TOCTTOU vulnerability. In order to choose the best steps, we did experiments under different steps and recorded the EPT violations and actual hypercalls. We test steps from 1 to 10, each one runs 10 times and calculate the average value. The rate equals the number of EPT violations divided by the number of hypercalls. The result is shown in Fig. 5. When the steps is set to 5, it is most economic as the rate of hypercalls consists of EPT violations reaches 60 %. And the rate won’t change much when setting larger steps, on the contrary the HVI-EPT will miss a lot of hypercalls between the interval.

An interesting result when setting steps is that when the step is 4, the rate is steadily 50 % during the 10 repeated tests, with variant to be only 6.07E−08. Even though it’s not the step with largest rate, it definitely is the most steady one. Consult to our analysis in “Hypercalls Intercepting”, every hypercall entry consists at least four instructions. So setting the step to 4 can reduce the repetition of VMEXITs caused by the code in the same hypercall entry. And after the 4 instructions are executed, the EPT protection is reset so as to monitor the next hypercall, thus avoid from missing hypercalls.

The performance of HVI-EPT-4 is tested in the “Analysis” section. Even though it still runs slower than HVI-BP, it is much better than the original HVI-EPT, which is too slow to be able to use.

## Analysis

The configuration of our experiments are shown in Table 1.

**Table 1:** Experiments configurations

Hardware: Intel Xeon CPU E5-2603 v3 1.60 GHz, 32GB DDR-4 memory, and 4 TB disk	
L0: Xen-4.6.0, 32GB memory, 4 TB disk	
L0 dom0: Ubuntu 14.04, kernel 3.19.0-24-generic, 8GB memory, 1 vcpu	
L1: Xen-4.4.1, 2GB memory, 10GB disk	
L1 dom0: Ubuntu 14.04, kernel 3.13.0-24-generic, 1GB memory, 1 vcpu, 5GB disk	
L1 domU: Ubuntu 14.04, kernel 3.13.0-24-generic, 512M memory, 1 vcpu, 5GB disk	

### Database preparation

**Fig. 6:**
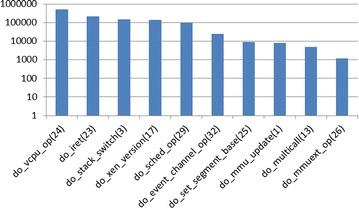
Top ten hypercalls (the *numbers in the brackets* represent the hypercall number)

**Fig. 7:**
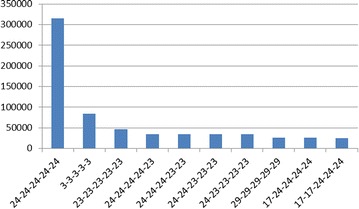
Top ten hypercall slices. *Each slice* is expressed as the combination of the hypercall numbers, separated by short bar

First, we monitored the clean nested virtualization system where no malicious hypercalls are issued. The L1 is in its default configuration after installation, with a VM running apache server on it. The intercepted hypercalls will be used as training data to generate our benign hypercall database. During an hour’s continuous intercepting, we captured 1,167,264 hypercalls belong to L1. Figure 6 shows the top ten intercepted hypercalls which consist of more than 99 % of all hypercalls. Figure 7 shows the top ten hypercall slices when the size of slice is set to 5. The size is chosen according to experiences and primer conclusions of related works (Maiero and Miculan 2011; Forrest et al. 1996).

From the result we can see that most hypercalls are related with vcpu schedulers, memory updating and domain communications, which is reasonable as the L1 hypervisor need to schedule between different guests to optimize the utilization of resources. Do_vcpu_op is the most frequent single hypercall, because we only have one 1.6G CPU and the hypervisor need to keep scheduling it between L0 and L1, also dom0 and domUs. For slices, the top three are repetitions of the top three single hypercalls, namely vcpu_op (24), iret (23) and stack_switch (3). We can also find that vcpu_op and iret happen together very frequently, consists six of the top ten slices. And iret always happens after vcpu_op.

### Performance evaluation

Then, we use three different ways to intercept hypercalls, including two methods of us, namely that based on EPT protection (HVI-EPT, for short) and hypercall breakpoint injection (HVI-BP, for short). Another controller method is Xentrace, a logging tool companioned Xen. Xentrace installs lots of static probes in the source code of Xen to capture trace buffer data from Xen, such as reasons, counts and the time of VMEXIT events, including hypercalls. It will generate about 19M raw data in just one minute during our test. Besides, as it directly communicates with the hypervisor to get the related information, we can only run it in L1 dom0 to get the hypercalls of L1 guests, which breaks the premise that no agents should run in the L1. By comparison, our HVI system, neither HVI-EPT or HVI-BP, both can obtain the hypercall information of all L1 guests from outside of L1 and make no modification to the L1 hypervisor.

To analysis their performance quantitatively, we logged the hypercalls and resource costs of the three methods during one minute, which are shown in Table 2. From the table we find that our HVI methods get less hypercalls than Xentrace in the same period of time, corresponding more CPU cost. This is because our HVI methods need to handle VMEXITs caused by #BP or EPT violations. As the performance cost increases, the number of hypercalls got executed in the same period of time decreases. However, it’s worth noting that the actual affect to L1 is very small, as CPU-L0 only slightly increases from 0.4 to 2.5 %. And the memory cost has no change between the three methods as our methods mainly change the execution path instead of memory consumption. The log size of Xentrace is apparently larger than our methods and is almost impossible to use in real-time monitoring.

From Table 2 we can also find HVI-EPT gets more hypercalls and costs more resources than HVI-BP . HVI-EPT monitor hypercalls in a page size, which would cause multiple EPT violations for one single hypercall as we analyzed in “Hypercalls Intercepting”. Actually, many of them are repeated records. HVI-BP only injects 0xCC at the start base of every hypercall entry, thus avoiding repeated VMEXITs and reducing performance cost.

**Table 2:** Performance comparison without workload

Metrics	Xentrace	HVI-BP	HVI-EPT
Hypercalls	12,992	3963	6708
CPU-L0	1.9 %	4.8 %	16.5 %
Mem-L0	25.1 %	25.1 %	25.1 %
CPU-L1	0.4 %	0.5 %	2.5 %
Mem-L1	6.3 %	6.3 %	6.3 %
Log-size	16M	14K	255K

To test the HVI system’s performance in practical usage, we did a set of workload tests using Apache ab tool, bonnie++ and unixbench. Apache ab tool is widely used to test the handle ability of web sites. We separately send 100, 1000 and 10,000 packages in concurrency of 10 to the website on L1 domU and compare the time to accomplish. Bonnie++ is a popular benchmark used to test the performance of disk operations. Unixbench is a comprehensive test bench to test the overall system performance.

**Table 3:** Time cost of Apache ab test (in s)

Number of requests	100	1000	10,000
Clean	1.870 (1)	18.645 (1)	148.904 (1)
HVI-BP	4.864 (2.60)	74.360 (3.99)	628.508 (4.22)
HVI-EPT-4	10.239 (5.48)	127.987 (6.86)	1318.938 (8.86)
HVI-EPT	64.644 (34.57)	879.799 (47.19)	9467.052 (63.58)

The number in brackets is the rate compared with the clean state in the same column

The results in Table 3 show that the time costs increase more slowly than linearly with the number of network requests, but increase more quickly than linearly with the different methods in the first column. Actually, if there are too many requests during a short period of time, the anti-DOS policy of the requested website would block our IP. So we must control the number of the requests under a threshold. We have repeatedly test ab for 10 times and used the average. So the results should be representative. Overall, HVI-BP and HI-EPT-4 is in the same magnitude with the original clean state, while HI-EPT is one magnitude slower. This result is in consistent with our former analysis.

**Fig. 8:**
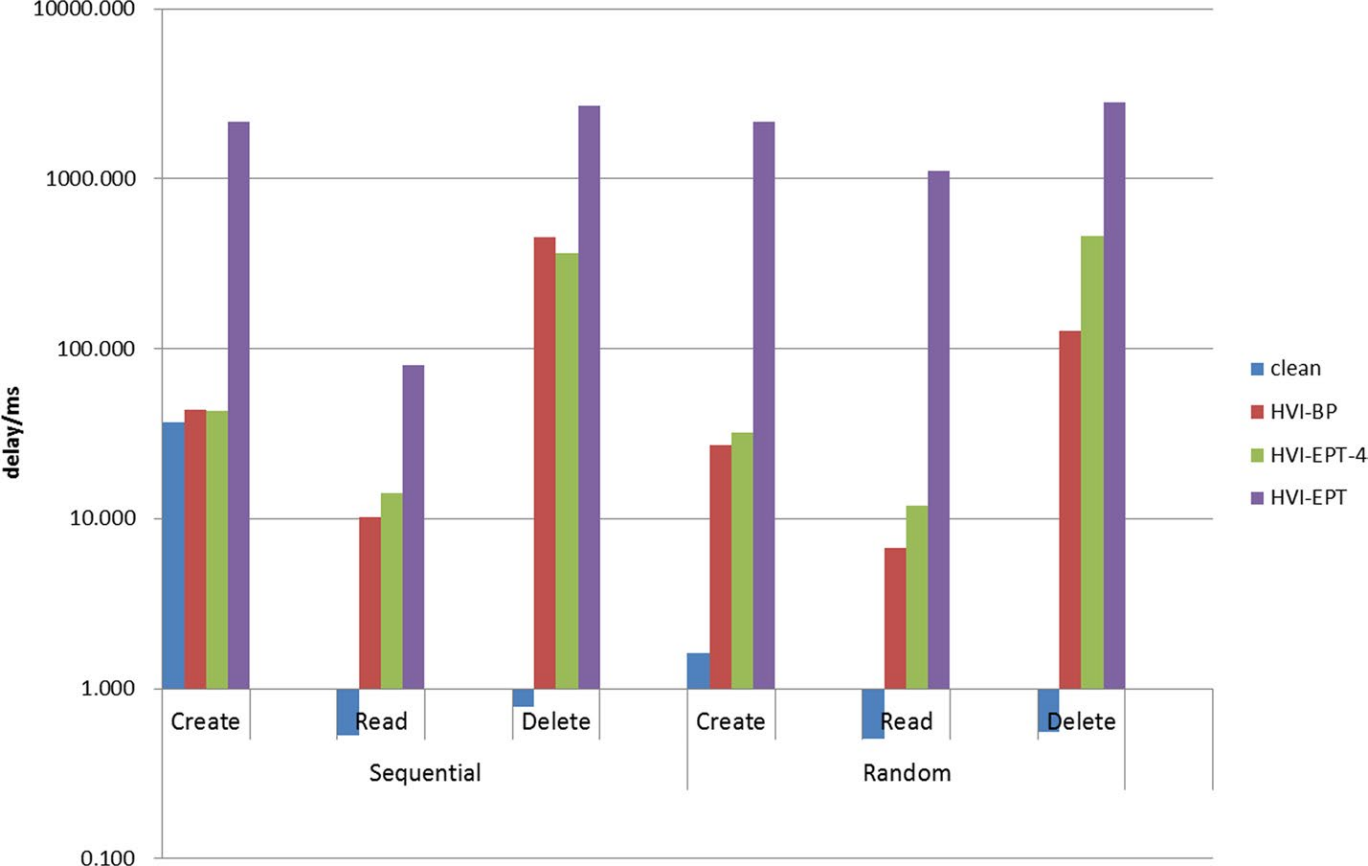
Performance of bonnie++

Figure 8 are the results of bonnie++, from which we can see that HI-EPT is very performance costing and the slowest in all the tests. Overall, HI-EPT-4 improves the performance of HI-EPT at about one magnitude and is close to the level of HI-BP. HI-BP works better in sequential operations than random operations, and better in file creation than file deletion. The read operation of HI-BP is about one magnitude slower than the clean state and the delete operation is about two magnitude slower. While to read operations, the sequential create operation of HI-BP has nearly the same performance as the clean state, while the random create operation are nearly one magnitude slower.

**Table 4:** Scores of unixbench

Benchmarks	Clean	HI-BP	HI-EPT-4
Dhrystone 2 using register variables	1578.5	1170.0	949.5
Double-precision whetstone	431.1	396.6	376.2
Execl throughput	154.1	1.5	0.8
File copy 1024 bufsize 2000 maxblocks	297.1	3.4	1.9
File copy 1024 bufsize 2000 maxblocks	183.1	2.1	1.1
File copy 256 bufsize 500 maxblocks	708.1	9.3	5.2
File copy 4096 bufsize 8000 maxblocks	119.9	1.0	0.6
Pipe throughput	68.6	0.8	0.4
Process creation	117.9	1.5	0.8
Shell scripts (1 concurrency)	401.6	4.1	2.0
Shell scripts (8 concurrency)	376.1	2.6	0.9
System call overhead	69.7	0.4	0.2
Overall score	242.5	5.0	2.8

Table 4 are the results of Unixbench, which show a huge difference between the time to accomplish the task. Dhyrstone and Whetstone are used to test the integer and floating point operations, which are close to the clean state. However, the performance of I/O intensive operations including File Copying and Pipe Throughput, and privileged operations including Execl, Process Creation, Shell Scripts and System Call all degrades sharply compared with the clean state. The overall scores of unixbench declines sharply, which indicates a real-time monitoring might slow down the legal program running on VMs. Although we might cheat the VM by modifying the showing time, users’ experience will uncover the existence of monitoring. This is indeed a limitation as a tradeoff between the usability and security, but we will improve it with new hardware support such as #VE and VMFUNC, which will be discussed in further works.

### Hypercall-based attacks detection

Hypercall-based attacks are simulated with HInjector. The attacks in Table 5 are based on real disclosed Xen vulnerabilities such as CVE-2012-3495, CVE-2012-5513, CVE-2012-5510, etc. The HInjector constructs the hypercalls by reverse engineering the existing patches to these vulnerabilities. For example, the official description of CVE-2012-3495 is “The *physdev_get_free_pirq* hypercall in $\backslash arch \backslash x86 \backslash physdev.c \backslash$ in Xen 4.1.x and Citrix XenServer 6.0.2 and earlier uses the return value of the *get_free_pirq* function as an array index without checking that the return value indicates an error, which allows guest OS users to cause a denial of service (invalid memory write and host crash) and possibly gain privileges via unspecified vectors”. HInjector simulates it by setting the value of its first parameter to 23 (namely *get_free_pirq*) and the value of the field type of its second parameter (struct *physdev_get_free_pirq*) to 1, then executed the *hypercall_physdev_op* 100 times. Besides these uncovered vulnerabilities, we also use some manual crafted random hypercall sequences to test the ability of HVI in detecting anomaly hypercalls. These test instances are listed in Table 5.

**Table 5:** Hypercall-based attacks by HInjector

Attacks	Hypercalls	Repeats	Args
CVE-2012-3495	physdev_op	100	cmd=23(physdev_get_free_pirq ->type=1)
CVE-2012-5513	memory_op	1	cmd=11(xen_mem_exchange{in{ nr_extents=1, extend_order=0, extent_start=1, domid=2}, out{nr_extents=16, extend_order=1, extend_start=1844660388597701000, domid=2})
CVE-2012-5510	grant_table_op	100*2	cmd=8(gnttab_set_version ->version=1/2)
example1	grant_table_op	100*2	cmd=8(gnttab_set_version ->version=1/2)
example2	get_debugreg	2	reg=1,3,4
example3	get_debugreg set_trap_table	2+1	reg=1,3,4 trap_info{flags=10, cs=2,address=3}
default	grant_table_op	100*2	cmd=8(gnttab_set_version ->version=1/2)

**Fig. 9:**
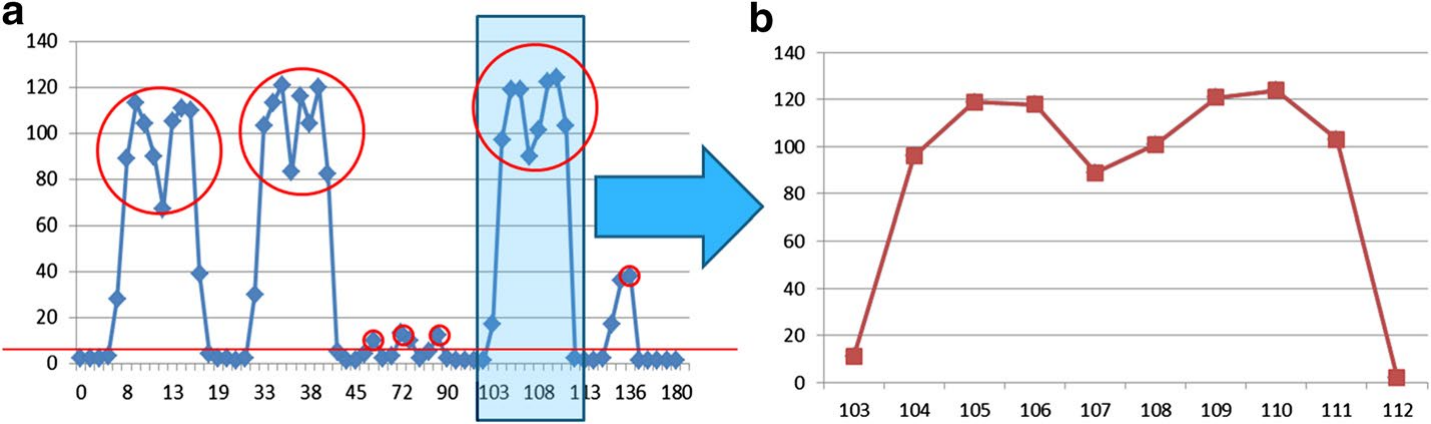
**a** Abnormal hypercall counts in a period of 3 min; **b** abnormal hypercall counts of a specific process during 103–112 s

Figure 9a is the result of orderly executing default, example1, example2, example3, CVE-2012-5513, CVE-2012-5510, CVE-2012-3495 in a period of 3 min. We can see that there are seven abnormal peaks corresponding to these attacks which is easy to observe from the real-time statistic chart. So in order to detect all these attacks, we just need to set the threshold to 5.

We need to clarify that HVI is used to detect hypercall attacks that use a sequence of crafted hypercalls. For those hypercall-based attacks that only use one hypercall with crafted arguments, our system can also partially detect it as one abnormal hypercall would cause multiple abnormal hypercall slices. For example, a normal hypercall sequence is 1-2-3-4-5-6-7-8. When there is an abnormal hypercall 9 happened between 4 and 5, it would generate five abnormal slices of length 5, including 1-2-3-4-9, 2-3-4-9-5, 3-4-9-5-6, 4-9-5-6-7 and 9-5-6-7-8. And that’s why choosing five as the threshold can filter out attacks in the experiment. Some hypercall-based attacks which only modify the arguments of a hypercall during normal hypercall sequences cannot be detected using HVI. But we can record the hypercall arguments for later forensic and investigation.

As Fig. 9a is a comprehensive overlook at the whole virtual machine, we’d like to further identify which process caused the abnormals. Take the peak during 103–113 s as example, firstly we correspond each abnormal hypercall to a process by translating the CR3 value to pid, then we extract the most frequent process which is the biggest suspect. Finally, we analysis the distribution of abnormals of this process, whose result is in Fig. 9b. Form Fig. 9b we find that the distribution of abnormals of this process is consistent with that of the VM during 103–113 s period, which validates its responsibility in causing the VM abnormal.

**Fig. 10:**
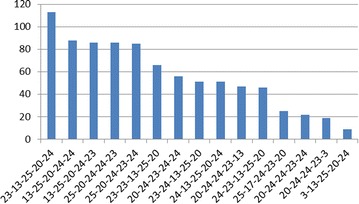
Top 15 abnormal-sequences of the suspicious process

After confirming the suspect, we would ulteriorly investigate the reason of these abnormals. So firstly we classify the abnormals of the suspicious process and we find the most frequent abnormal hypercall sequences as shown in Fig. 10. We can find that every abnormal hypercall sequence in the top 15 consists *hypercall grant_table_op* (20), so it might be a hypercall-based attacks by replaying hypercalls with crafted arguments. As the hypercall arguments are stored in registers %rax, %rbx, %rcx, %rdx, %rsi and %rdi when it’s executed, we extract the arguments of hypercall *grant_table_op* when it triggers the #BP in the interrupt callback function. And we find %rdi is always 8 when *grant_table_op* is executed, which identifies the way attackers craft hypercall-based attacks. And what remains is to find out why this crafted hypercall sequences are used by attackers and if there is any problems in the code or logic design. This would helps in timely patching the vulnerabilities and improving the security of hypervisor design.

To detect future attacks, we can use the detected abnormal hypercall sequences as the malware signature. We can also analysis the abnormal hypercalls to obtain the abnormal arguments and add them into the database as signatures.

## Discussion and future work

### Performance issue

Even though we take measures including setting intervals between EPT protection, using #BP instead of #EPT, our HVI system still has large performance impact on the VMs when there is workload as shown in Table 3. Firstly, we thought it might be the conflict between the L0 and L1 as they scramble same CPU. So we separate the CPU resource by binding L0 and L1 on different vCPUs. After that, we monitor the resource consuming when HVI works and find that the L0 runs at 100 % vCPU while L1 costs less than 10 % vCPU. So we conclude that the performance degradation is due to the delay of L0 by handling of VMEXITs.

During VMEXITs, the L0 has to record hypercalls information to file/console, analyze the hypercall sequences to decide if they are malicious, reset the events to continuously monitoring hypercalls. Only after all these jobs are done that the new instruction of the L1 will be proceeded. So the L1 is actually always in a pause state of waiting for the handling results of L0. To improve that, we have three approaches.

The first one is by reducing the jobs L0 has to handle during VMEXITs. We can just record the hypercalls information into files and deliver it to the analyzer for offline analysis. This is sufficient for hypercall-based attacks detection or forensics, but would lose the ability of real-time defense and prevention. Another time consuming work is resetting the monitoring events. When an #BP event happens, we should first remove the injected breakpoints and write back the original value where breakpoint is injected, then set a single step event to let the original instruction get executed and reset the breakpoint after it is executed. If we can directly simulate the #BP when VMEXIT, we can avoid from repeatedly removing and resetting breakpoints. And the emulation can be done using hvm_emulate_one function of Xen hypervisor, which will be remained as our future works.

The second is by putting the monitor in L1 and protect it form compromising by malware on L1 with hardware features such as #VE and VMFUNC. The EPT violation and #BP both cause the VM exits into VMX root mode which will introduce a lot of performance cost. However, when #VE is available and enabled, such an virtualization exception will induce an guest exception in VMX non-root mode, and thus being handled directly in guest OS to reduce the performance cost. VMFUNC is a new VMX instruction, which can only be executed in guest OS (VMX non-root mode). So it is very suited to handle the #VE. However, the VMFUNC is a relative new instruction and currently has no practical application. So it needs further exploring in the way utilizing this new feature. The #VE and VMFUNC feature are very similar to the latest Intel SGX feature, which provides an enclave for credential applications running and disabling other privileged software layers from accessing this enclave (Anati et al. 2013). So the new HVI can keep itself secure in L1 while in the same time monitor L1.

The third one is avoiding the L1 from being paused while L0 is handling the specific VMEXITs by #BP and #EPT. Then the only thing we need to do is sending the useful information to L0 while this VMEXITs happen and there is no need to stop the execution of L1. However, this would violate the design principle of Xen because we can’t differentiate normal VMEXITs with that caused by HVI until we obtain the control of L1 from L0. So we can’t just let these VMEXITs events go as they might be dangerous operations. The only way to do this is by modifying Xen code and adding logs to functions which might cause VMEXITs so as to capture related information. Actually, this is exactly what Xentrace does. As it violates our design principle of no modification to hypervisors, we are not going to take this method.

### Anti-detection of L0

As nested virtualization is used to implement HVI, it is worth to note that the L0 might be detected. As nested virtualization is not normal in practical usage, it can be inferred as a signal of monitor’s existence. Just as the existence of virtualization may indicate the existence of honeypot, malware or attackers may hide their existence by keeping silent. In order to avoid L0 from being detected, we studied the currently detection methods and provides some solutions to deal with them.

The easiest detection method is by counting the time to complete a specific task and comparing it with that on clean system. However, the time can be changed by the hypervisor before it is printed on the screen, so it’s unreliable. But by using remote timer such as NTP, the user or attacker might find there is weird clock skew (Garfinkel et al. 2007). However, the time delay could be introduced by L1 hypervisor, instead of that by L0. As long as we keep a persistent L0 monitoring, it would be difficult for the attacker to judge whether the delay is caused by L1 or L0.

Other existing hypervisor detection methods mainly aim at detecting the top layer hypervisor, with nested hypervisor being ignored. For example, by assigning different values to %eax and issuing the CPUID instruction, it is able to detect the presence of Xen. However, the CPUID instruction would also cause VMEXIT and can be intercepted by the bottom hypervisor. What’s more, with the development of nested virtualization support, most combination of Xen, VMware and Hyper-v can be supported, some of which even support multiple layer nesting. So just detecting the existence of the first layer hypervisor is not enough.

To detect nested hypervisor, Actaeon is designed (Graziano et al. (2013)). The basic idea of Actaeon is through searching feature field values in virtual machine control structure (VMCS) and deciding the relationship between hypervisors according the layout of different VMCS. VMCS is the memory structure that contains information for keeping the state of the system when it switches from hypervisor to virtual machines and vice versa. Actaeon relies on accessing to raw physical memory, which is impossible for a VM user without physical access privileges as we assumes.

Another interesting work trying to detect the existence of hypervisor is also named “hypervisor introspection” (HI) (Wang et al. 2015), but the concept is different with our HVI. HI uses the “VM suspend side-channel” to detect the existence of passive VMI, thus an attacker can execute the malicious activity in the interval of monitoring. However, the author also clearly states that the HI method could not defend active introspection which HVI does, and it may also fail when the passive VMI randomizes monitoring interval.

SOK (Jain et al. 2014) discussed possible attacks to fool VMI tools by guest kernel manipulating, including DKSM (Bahram et al. 2010). It also proposed solutions such as paraverification and SGX to remove the guest OS from TCB. Paraverification requires modification to the guest OS, just as paravirtulization. SGX needs modification to the current hardware and related products are already released in 2015 October. So in the future we might just implement VMI or HVI in SGX enclaves, thus protecting it from being tampered by untrusted OS or untrusted hypervisor. Our HVI identifies the PID from the cr3 register, which assumes a measure of trust in the guest OS. So the cr3 value might not consistent with that of process as the guest might be malicious, but this problem can be solved by cross-view validation (Jones et al. 2008).

Nevertheless, absolutely avoid of nested virtualization being detected is hard to implement and might affect the usability, just as virtualization detection and anti-detection (Garfinkel et al. 2007). But by utilizing anti-detection methods provided in this discussion, we can at least improving the difficulty of L0 being detected. What’s more, the detection of hypervisor doesn’t mean the discovery of nested hypervisor, as long as we keep a persistent active monitoring.

### Application scenario

Our system is mainly designed to detect hypercall-based attacks aimed at compromising the hypervisor. But its application is not limited in this case. Other possible application scenarios include:

*Hypercall access control* Our system can be easily extended to an access control system based on hypercall. As we can intercept hypercalls of specific guest, we can disable it or enable it based on the policy that defines which guest can access which hypercall. We can also accomplish small granularity access control considering the arguments and return values of hypercalls. This is similar to what XSM does, but more flexible than XSM. XSM need to modify Xen source code to inject hooks at the hypercall functions, which is hard to update when new hypercall is introduced. While our solution only need to know the new virtual address of the new hypercall, which can be easily obtained from hypervisor debug files.

*Honeypot for hypervisors* As new editions of hypervisors are released very frequently, especially those open-source ones such as Xen and KVM. Enterprises and individuals can both obtain and modify them easily according to their only requirements, which provides a chance for the attackers. The attackers can modify the open-source hypervisor and add malicious hypercalls then publishing them on various kinds of websites to induce others to download. The latest accident of APPLE’s Xcode is such a case (Xiao 2015). So it is crucial that we have a controllable environment to monitor the behavior of various versions of hypervisors from various sources. The HVI architecture which utilizes nested virtualization and EPT protecting technology meets the demands very well. To treat hypervisor as a virtual machine, we can easily extend the traditional virtual honeypots to honeypots for hypervisors. But the information acquisition method is different as the monitored object is different. The hypercall intercepting technology of our system provides one solution. Further information obtaining, such as events and network behaviors leaves as an open issue.

## Related work

A lot of works have been done to ensure the security of hypervisor, including hypervisor integrity verifying (Azab et al. 2010; Wang and Jiang 2010), hypervisor access control (Le 2009), hypervisor rootkit detection (Gebhardt et al. 2008), and trust hypervisor (Neisse et al. 2011). These methods greatly enhanced the robust of hypervisors. However, they are more concentrated on preventing hypervisors from being attacked, rather than detecting attacks to hypervisors.

Our main goal is to monitor the hypercalls to detect malicious hypercall-based attacks to hypervisor while in the same time keep the monitor from being detected. To intercepting hypercalls for analyzing VM behavior, XSM use hypercall hooks (Coker 2006). The hook method has benefits of high performance and small granularity. However, as the same problem of syscall hooking, hypercall hooking would undergo the problem of being detected and attacked. Besides, it requires modifying the Xen hypervisor to implement it, which violates our assumptions in “Design” section.

From the perspective of security design, the hypervisor is the most privileged software layer in cloud platform. In order to monitor it’s behavior, we need to implement our monitoring tools in hardware. There have been similar works which using console, firewire, and other PCIE interfaces to debug or do memory forensic to hypervisor (Carrier and Grand 2004). They need modification to the hardware and are hard to implement. Moreover, the System Management Mode (SMM) is also used to protect the hypervisor (Azab et al. 2010). Although it has better isolation and are more stealthy than the hooking solution, the SMM may also be exploited to subvert the hypervisor (Rutkowska and Wojtczuk 2008).

There are aslo works related with detecting hypercall-based attacks, such as Collabra (Bharadwaja et al. 2011), Xenini (Maiero and Miculan 2011), C2Detector (Wu et al. 2014), MAC/HAT (Le 2009), RandHyp (Wang et al. 2012). Collabra is a collaborative hypercall-based IDS. Collabra gives each hypercall an anomaly score according to related elements. By comparing the anomaly scores with a threshold, Collabra divides hypercalls into two categories as normal and anomalous. This method is relatively unreliable since both legitimate and malicious behavior could cause a suspicious hypercall with high anomaly scores. In contrast, we use anomaly-based methods to analyze the hypercalls and find anomalous behavior, which is more dependable. Xenini patches the Xen to trace calls so as to detect intrusions to VMs while our system is used to detect intrusions to hypervisor without modify the hypervisor. C2Detector install hooks inside the hypervisor to monitor all the hypercalls so as to detect covert channel, but it will break the integrity of the hypervisor and introduce potential new vulnerabilities. By contrast, our system use an out-of-box way to monitor the execution of hypervisors, which is more flexible and secure. MAC/HAT uses MAC (Mandatory Access Control) and HAC (Hypercall Address Table) to authenticate and control the access of hypercalls. But it doesn’t detail the problem of how hypercalls are intercepted and only focuses on hypercall integrity protecting instead of hypercall-based attacks detection. RandHyp is closest to ours, which adopts randomization technique to randomize trust hypercall’s number and arguments, then recover them in the hypervisor. However, it can only be used to detect illegal hypercalls which are added by the intruders, instead of attacks using legal hypercalls sequence or crafted arguments.

Other related works such as in-box detecting of malware to improving the efficiency (Lin et al. 2011) and remote attestation to ensure the trustworthy of guest kernel (Mei et al. 2012), combined with our work, would greatly improve the overall security of the cloud platform.

## Conclusion

In this paper, we introduced HVI, a nested hypervisor introspection framework. HVI is used to intercept hypercalls and detect hypercall-based attacks. To achieve that, we rely on CPU features such as EPT and #BP to trigger VMEXIT when hypercalls happen. HVI is able to detect hypercall-based attacks including crafting arguments, crafting hypercall sequences, and malicious new hypercalls. What’s more, HVI doesn’t modify the monitored hypervisor or install any agents in the guests, which is very suitable for building hypervisor sandbox and hypercall access controlling.
